# Establishing Classification Tree Models in Rheumatoid Arthritis Using Combination of Matrix-Assisted Laser Desorption/Ionization Time-of-Flight Mass Spectrometry and Magnetic Beads

**DOI:** 10.3389/fmed.2021.609773

**Published:** 2021-02-24

**Authors:** Dan Ma, Nana Liang, Liyun Zhang

**Affiliations:** ^1^Department of Rheumatology, Shanxi Bethune Hospital, Shanxi Academy of Medical Sciences, Shanxi Bethune Hospital Affiliated to Shanxi Medical University, Taiyuan, China; ^2^First Hospital/First Clinical Medical College of Shanxi Medical University, Taiyuan, China

**Keywords:** rheumatoid arthritis, MALDI-TOF-MS, weak cationic exchange magnetic beads, biomarkers, classification tree model

## Abstract

**Background:** There is no simple method for early diagnosis and evaluation of rheumatoid arthritis (RA). This study aimed to determine potential biomarkers and establish diagnostic patterns for RA using proteomic fingerprint technology combined with magnetic beads.

**Methods:** The serum protein profiles of 97 RA patients and 76 healthy controls (HCs) were analyzed by matrix-assisted laser desorption/ionization time-of-flight mass spectrometry (MALDI-TOF-MS) with weak cationic exchange (WCX) magnetic beads. Samples were randomly divided into training (83 RA patients and 56 HCs) and test sets (14 RA patients and 20 HCs). Patients were classified according to their Disease Activity Score: in remission, *n* = 28; with low disease activity, *n* = 17; with moderate disease activity, *n* = 21; with high disease activity, *n* = 31. There are 44 RA patients alone, 22 RA patients with interstitial lung disease (RA-ILD), 18 RA patients with secondary Sjögren's syndrome (RA-sSS), 6 RA patients with osteonecrosis of the femoral head (RA-ONFH), and 7 RA patients with other complications. Eleven patients were treated with etanercept only for half a year, after which their serum protein profiles were detected. The proteomic pattern was identified by Biomarker Patterns Software, and the potential biomarkers for RA diagnosis were further identified and quantified by enzyme-linked immunosorbent assay.

**Results:** The diagnostic pattern with four potential protein biomarkers, mass-to-charge (m/z) 3,448.85, 4,716.71, 8,214.29, and 10,645.10, could accurately recognize RA patients from HCs (specificity, 91.57%; sensitivity, 92.86%). The test set were correctly classified by this model (sensitivity, 95%; specificity, 100%). The components containing the four biomarkers were preliminarily retrieved through the ExPasy database, including the C-C motif chemokine 24 (CCL24), putative metallothionein (MT1DP), sarcolipin (SLN), and C-X-C motif chemokine 11 (CCXL11). Only the CCL24 level was detected to have a significant decrease in the serum of RA patients as compared with HCs (*p* < 0.05). No significant difference was found in others, but a decreasing trend consistent with the down-regulation of the four biomarkers detected by MALDI-TOF-MS was observed. The diagnostic models could effectively discriminate between RA alone and RA with complications (RA-ILD: m/z 10,645.10 and 12,595.86; RA-sSS: m/z 6,635.62 and 33,897.72; RA-ONFH: m/z 2,071.689). The classification model, including m/z 1,130.776, 1,501.065, 2,091.198, and 11,381.87, could distinguish between RA patients with disease activity and those in remission. RA with low disease activity could be efficiently discriminated from other disease activity patients by specific protein biomarkers (m/z 2,032.31, 2,506.214, and Z9286.495). Two biomarkers (m/z 2,032.31 and 4,716.71) were applied to build the classification model for RA patients with moderate and high disease activities. Biological markers for etanercept (m/z 2,671.604064, 5,801.840579, 8,130.195641, and 9,286.49499) were observed between the responder (*n* = 7) and non-responder groups (*n* = 4) (*p* < 0.05).

**Conclusion:** We successfully established a series of diagnostic models involving RA and RA with complications as well as assessed disease activity. Furthermore, we found that CCL24 may be a valuable auxiliary diagnostic indicator for RA. These results provide reference values for clinical practice in the future.

## Introduction

Rheumatoid arthritis (RA) is a chronic systemic autoimmune and inflammatory disease characterized by synovitis and vasculitis, which lead to the destruction of cartilage, joint deformation, loss of joints function, and systemic organ damages, and it affects approximately 0.5–1% of population (1). Pulmonary involvement is a common extraarticular manifestation of RA (2), particularly interstitial lung disease (ILD), which occurs in 1–58% of RA patients and has significant effect on morbidity and mortality (3, 4). In addition, both secondary Sjögren's syndrome (sSS) (5, 6) and osteonecrosis of the femoral head (ONFH) in RA have a pooled prevalence of 19.5 and 5.75–53.8%, respectively (7, 8).

Diagnosis of RA according to the American College of Rheumatology (ACR) criteria in 1987 is based on clinical symptoms, presence of rheumatoid factor (RF), and imaging tests; however, such criteria are not suitable for early-stage RA patients. Compared with the 1987 ACR diagnosis guideline, the sensitivity of the ACR/2010 European League Against Rheumatism (EULAR) classification standard is higher mainly due to the addition of anti-citrullinated protein antibodies. However, the specificity of anti-citrullinated protein antibodies in RA is only 60–75%, which suggests low diagnostic efficacy (9). A population-based incidence study revealed that the incidence of RF-negative RA has significantly increased and RF-positive RA has significant decreases (10). These can lead to difficulty in early diagnosis. Furthermore, disease activity is evaluated by the Disease Activity Score (DAS) tool and others, which are complex, inconvenient, or maybe not suitable for general clinical practice. High-resolution computer tomography, which is used for diagnosing early possible complications such as ILD (11), is expensive and has radiation risk (12). Although magnetic resonance has been demonstrated to be a useful imaging test in diagnosing ONFH, it is also expensive (13, 14). Meanwhile, due to low sensitivity and specificity of anti-Ro and anti-La antibodies and considering the invasiveness of lip biopsy, diagnosis of early RA-sSS is difficult (15, 16). Therefore, there has been increasing interest in identifying specific and powerful biomarkers for both the diagnosis of RA, RA-ILD, RA-sSS, and RA-ONFH and the evaluation of disease activity in order to increase the diagnostic efficiency and early treatment.

Early intervention (≤ 3 months) using biologic agents was the strongest predictor of successful remission, as confirmed in clinical practice. However, clinical applications of biological agents have been limited by their expensive cost (17). Therefore, it is important to explore promising biomarkers to evaluate the potential clinical effects of etanercept and determine which patient would benefit the most, with the possibility of modulating treatment for RA patients who are not responding to the drug in order to reduce their economic burden.

Matrix-assisted laser desorption/ionization time-of-flight mass spectrometry (MALDI-TOF-MS) (18) is currently one of the most important and key proteomic technologies (19). It can be used to make high-throughput protein analysis of large samples, and it has high sensitivity, resolving capability, and reproducibility (20). Weak cation exchange (WCX) magnetic beads (21) use their large surface to capture proteins and small molecular peptides of interest (22). The use of MALDI-TOF-MS and WCX magnetic beads, especially combined with bioinformatics tools, is appropriate for setting up the classification tree model to assist in preliminary biomarker discovery (23).

Many researchers have already applied MALDI-TOF-MS to generate protein fingerprints and build serological classification tree models in certain diseases, including rheumatic diseases [early RA (24), RA (25), systemic lupus erythematosus (26), and SS (27)] and other diseases (28, 29), and these were highly effective in discriminating patients and controls (30). However, these studies only built the diagnostic model for RA patients, but they did not have database retrieval, and some studies lacked further validation for the classification tree models. This study aimed to detect a series of specific proteomic diagnostic model for RA, RA-ILD, RA-sSS, and RA-ONFH as well as the potential biomarkers to distinguish the RA disease activity and to identify etanercept's clinical effect using proteomic fingerprint technology (MALDI-TOF-MS) combined with WCX magnetic beads. For the potential protein for diagnosis of RA, we made a further preliminary retrieval through the ExPasy database and verified these biomarkers by enzyme-linked immunosorbent assay (ELISA).

## Methods

### Patients and Healthy Controls

A total of 173 serum samples were collected in our study from May 2015 to July 2017 at Shanxi Bethune Hospital. The study population included 97 RA patients (RA alone, *n* = 44; RA-ILD, *n* = 22; RA-sSS, *n* = 18; RA-ONFH, *n* = 6; RA with other complications, *n* = 7) and 76 healthy controls (HCs). All patients were diagnosed using the 1987 ACR or the 2010 ACR/EULAR criteria. All patients were classified according to their DAS, as calculated from the online Disease Activity Score-28 (DAS28) for Rheumatoid Arthritis with ESR tool based on 28 joints: in remission (DAS28 <2.6, *n* = 28), with low disease activity (2.6 < DAS28 ≤ 3.2, *n* = 17), with moderate disease activity (3.2 < DAS28 ≤ 5.1, *n* = 21), and with high disease activity (DAS28 > 5.1, *n* = 31). All ILD cases were diagnosed by high-resolution computer tomography of the chest. Meanwhile, all sSS patients were diagnosed based on the American-European Consensus Group classification criteria (2002) and the 2012 ACR classification criteria. ONFH diagnosis was based on the detection of marrow foci with decreased signals on T1-weighted images and the characteristic “double-line sign” on T2-weighted images. None of the patients had any active or latent bacterial, fungal, or viral infection at the time of enrolment. Eleven patients only received the same etanercept treatment after inclusion, and their clinical outcome was assessed at week 24. They never received anti-tumor necrosis factor therapy before then. Their ACR 20/70% improvement criteria (ACR20/70), which were used to determine the therapeutic effects of etanercept, C-reactive protein level, erythrocyte sedimentation rate, and presence of RF were evaluated. Finally, four non-responders (as defined by ACR20 negative) and seven responders (ACR70 positive) to etanercept at week 24 were evaluated in our research.

We use two cohorts to build and test the RA diagnostic model. Cohort 1 (training set) included 83 RA patients and 56 HCs to establish a serological classification tree mode to distinguish them. Cohort 2 (blinded testing set) included 14 RA patients and 20 healthy individuals to test the classification efficiency of this RA diagnosis model. The detailed clinical and demographic features of the study subjects are provided in Table 1. Furthermore, we used serum samples from RA patients (*n* = 22) and HCs (*n* = 22) for ELISA to verify the results of MALDI-TOF-MS.

**Table 1:** Clinical and demographic characteristics of patients and healthy controls.

**Characteristic**	**RA**	**HCs**
Age (years,‘x ± s)	56 ± 13	53 ± 12
Sex (male/female)	68\29	57\19
Disease duration (M (Q1, Q3), months)	84 (7,120)	—
Tender joint counts (M (Q1, Q3), numbers)	4 (0,24)	—
Swollen joint counts (M (Q1, Q3), numbers)	2 (0,24)	—
Erythrocyte sedimentation rate (M (Q1, Q3), mm/h)	42 (5,108)	—
C-reactive protein (M (Q1, Q3), mg/dL)	42 (5,108)	—
Rheumatoid factor, positive (%)	59 (71.1%)	—
Anti-CCP antibody, positive (%)	51 (61.4%)	—
DAS28ESR (′x ± s)	4.93 ± 1.30	—
Only Treatment of Recombinant Human Tumor Necrosis Factor-α ReceptorII:IgG Fc Fusion Protein for Injection(%)	11 (11%)	—

### Sera Collection and Preparation

Blood samples (4 mL) were collected and centrifuged at 3,000 rpm for 5 min at 4°C. All serum samples was divided and immediately stored at 80°C. The collection and analysis interval was within 3 months.

### Magnetic Bead–Based Sample Preparation for MALDI-TOF-MS

Serum samples were pretreated with magnetic beads. In brief, 10 μL of each serum sample was mixed with 20 μL of U9 (9 mol urea, 2% CHAPS) in a 0.5-mL EP tube. After incubating for 30 min at 4°C, the sample was diluted 1:40 by adding 370 μL of buffer (containing 50 mmol NaAC, pH 4.0). Then, 50 μL of WCX magnetic beads (50 mg/mL) was added to a polymerase chain reaction tube, which was placed in a magnet separator for 1 min, and the supernatant was carefully removed using a pipette. The magnetic beads were then washed twice with 100 μL buffer. A 100-μL diluted serum sample was carefully added and mixed with the activated magnetic beads by pipetting up and down several times; this was, incubated for 1 h at 4°C and washed twice with 100 μL buffer. After binding and washing, the bound proteins were eluted from the magnetic beads using 10 μL of 0.5% trifluoroacetic acid. Then, 5 μL of the eluted sample was diluted 1:2 in 5 μL of sinapic acid (50% acetonitrile + 0.5% trifluoroacetic acid), and 1 μL of the resulting mixture was aspirated and spotted onto 8 spots of prestructured sample support (Au-chip). After air-drying for approximately 5 min at room temperature, the protein crystal on the chip was detected by MALDI-TOF-MS (PBS IIc; Ciphergen Biosystems, Fremont, CA, USA).

### Enzyme-Linked Immunosorbent Assay

The serum concentrations were measured by ELISA using an ELISA kit provided by Xinqaun Company (Taiyuan, China) in accordance with the manufacturer's instructions.

### Data Analysis

The data analysis involved three stages: (i) peak detection and alignment; (ii) selection of differently expressed peaks among groups that may represent potential biomarkers of RA, RA-ILD, RA-SS, and RA-ONPH; (iii) data analysis using a decision tree algorithm.

Peak detection was performed using Ciphergen ProteinChip version 3.0.2 (Ciphergen Biosystems). The protein peaks with mass-to-charge (m/z) ranging from 2,000 to 50,000 were selected for analysis, whereas those with m/z ranging between 0 and 2,000 were eliminated from the analysis to avoid interference from adducts, artifacts from energy-absorbing molecules, and other possible chemical contaminants. Peak detection involved (i) baseline subtraction, (ii) mass accuracy calibration, and (iii) automatic peak detection. Using Biomarker Wizard version 3.1.0 (Ciphergen Biosystems), biomarkers that represent consistent protein peak sets across multiple spectra were generated. Baseline subtraction was performed on all spectra. The peak m/z of 4,901 was selected to normalize dimension. The settings for auto-detect peaks to cluster were as follows: first pass: signal-to-noise ratio, 5; minimum peak threshold, 10%; cluster completion: cluster mass window, 0.3%; second pass: signal-to-noise ratio, 2.

Statistical analysis was conducted using SPSS version 18 (IBM Corp., Armonk, NY, USA). Both parametric Student's *t* test and non-parametric Mann–Whitney *U* test were applied. Results were considered statistically significant if *p* < 0.05.

## Results

### Discriminating m/z Peaks Between RA and Control Subjects

Among the m/z 1,000–20,000 peak range, there were a total of 115 differential protein peaks between the RA and HCs groups. Of these peaks, 58 protein peaks statistically differed (*p* < 0.05), including 22 that were overexpressed in RA samples (*p* < 0.05).

### Establishment of the Serological Classification Tree Model for RA

#### RA Patients and HCs

**Figure 1:**
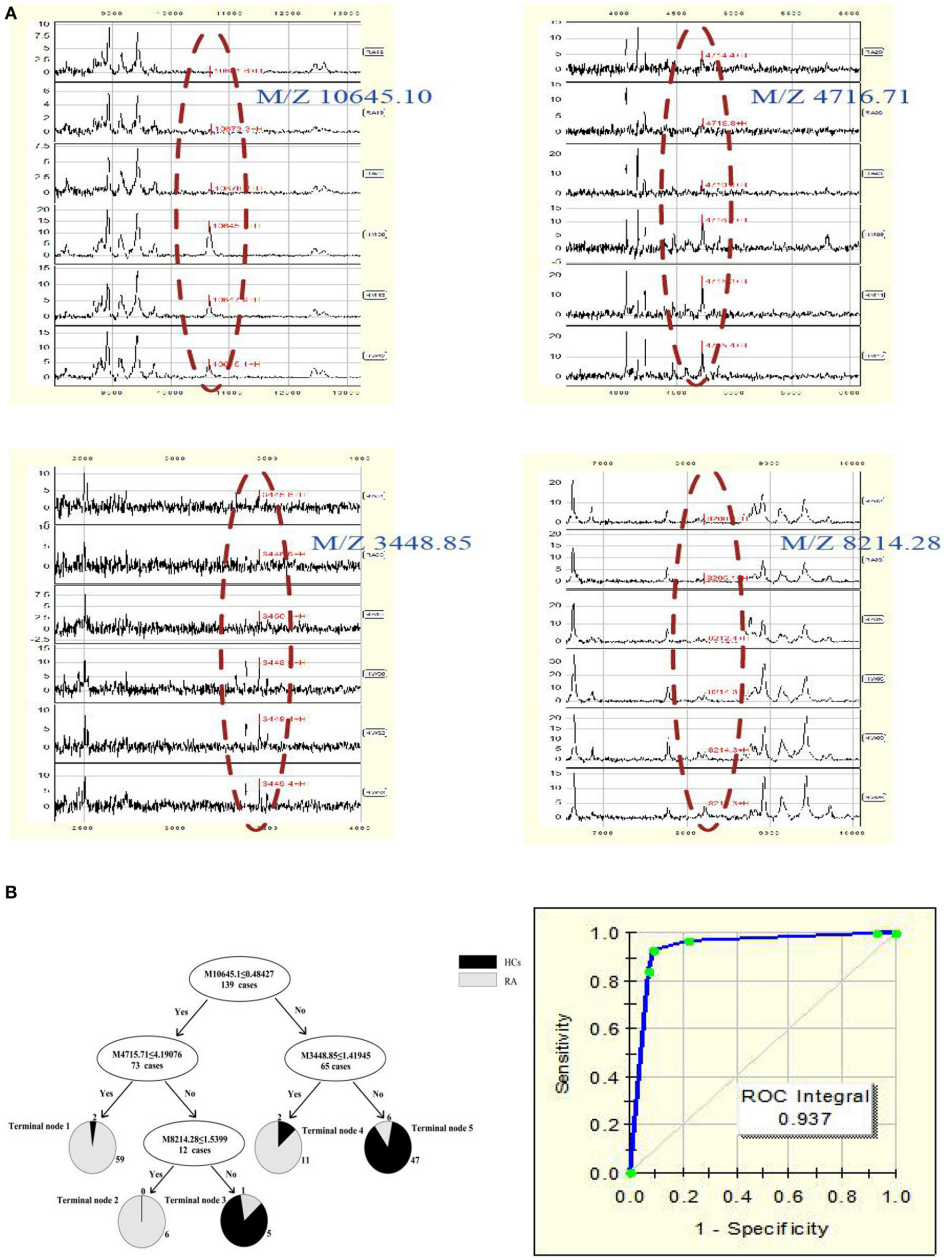
**(A)** Serum protein/peptide spectrum of RA patients and HCs. **(B)** Dot plots and ROC curve of selected biomarker candidates in patients with RA and healthy controls. The mass-to-charge value in the nodes was followed by lower or equal to intensity value. If the answer to the question in a node of the tree was yes, it proceeded down to the left node, otherwise it proceeded down to the right node. When samples allocated to terminal node 1, 2, and 4 were assigned as RA, whereas those classified into terminal node 3 and 5 were assigned as HCs.

A total of 22 protein peaks were used by the Biomarker Patterns Software (BPS) version 5.0 (Ciphergen Biosystems) to establish the most optimal classification tree based on the lowest error cost of misclassification (represented as relative cost of 0.462). The most optimal tree model consisted of m/z peaks 3,448.85, 4,716.71, 8,214.28, and 10,645.10 (Table 2), all of which were down-regulated in patients with RA compared with HCs (Figure 1A). The classical protein/peptide spectra of serum sample from RA patients and HCs are shown in Figure 1A. All 139 spectra of the training set were differentiated into five terminal nodes (Figure 1B). This classification tree had a specificity of 91.57% and a sensitivity of 92.86% for distinguishing between RA patients and HCs (Table 3). All 14 RA spectra and 20 HCs in the test set were correctly classified by this model, yielding a sensitivity of 95.00% and a specificity of 100% (Table 3). The integral of receiver operating characteristic curve (ROC) of this decision tree supplied by BPS version 5.0 was 0.937 (Figure 1B).

**Table 2:** Biomarker statistics for RA vs. HCs spectra in the decision tree classification.

**M/Z**	**RA (Mean ± SD)**	**HCs (Mean ± SD)**	***P***
10,645.10	10.39 ± 5.81	7.44 ± 5.06	0.002
4,716.71	2.27 ± 1.49	5.92 ± 4.25	0.000
3,448.85	2.37 ± 2.02	3.76 ± 2.10	0.000
8,214.28	1.39 ± 0.83	2.76 ± 1.66	0.000

**Table 3:** Prediction success of classification tree model for RA.

**Actual class(according decision tree classification class to clinical diagnosis)**	**Decision tree classification class**	
	**RA, *n* (%)**	**HCs, *n* (%)**
In the training set		
RA (*n* = 83)	76 (91.57)	7 (8.43)
HCs (*n* = 56)	4 (7.143)	52 (92.86)
In the blinded testing set		
RA (*n* = 14)	14 (100)	0 (0)
HCs (*n* = 20)	1 (5)	19 (95)

#### ELISA Validation

The components containing four targeted protein peaks of the RA diagnostic model were preliminarily retrieved through the ExPasy database. The proteins of m/z 10,645.10, 4,716.71, 3,448.85, and 8,214.28 might correspond to C-C motif chemokine 24 (CCL24; code: O00175), putative metallothionein (MT1DP; code: A1L3X4), sarcolipin (SLN; code: O00631), and C-X-C motif chemokine 11 (CCXL-11; code: O14625), respectively.

**Figure 2:**
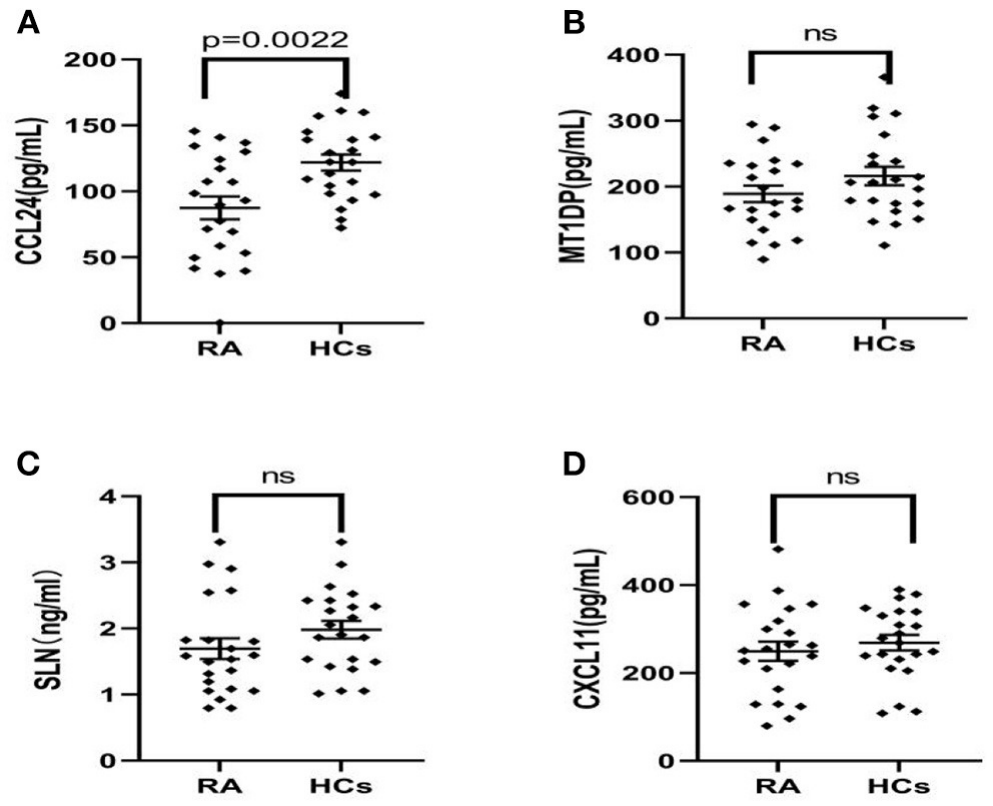
**(A–D)** ELISA results of CCL24, MT1DP, SLN, and CXCL11 in the serum of RA patients (*n* = 22) and health controls (*n* = 22), indicated the CCL24 level has a significantly decreased expression in the RA serum and other biomarkers had not a difference. Plots indicate individual protein level of each group. Data are presented as mean±SEM. ns, no significant.

Therefore, the above targeted biomarkers were further identified and quantified through ELISA. A significant decrease was observed in the CCL24 level in the serum of RA patients (75.12 ± 69.59 ng/mL) as compared with HCs (125.3 ± 41.9 ng/mL) (*p* < 0.05) (Figure 2A). No significant difference was found in MT1DP, SLN, and CXCL-11 between RA patients and HCs (Figures 2B–D). Remarkably, the ELISA results, i.e., the four biomarkers had lower levels in RA patients than HCs, were consistent with the MALDI-TOF-MS results.

### Establishment of the Classification Tree Model for Assessing Disease Activity in RA Patients

#### RA Patients With Remission and Disease Activity

**Figure 3:**
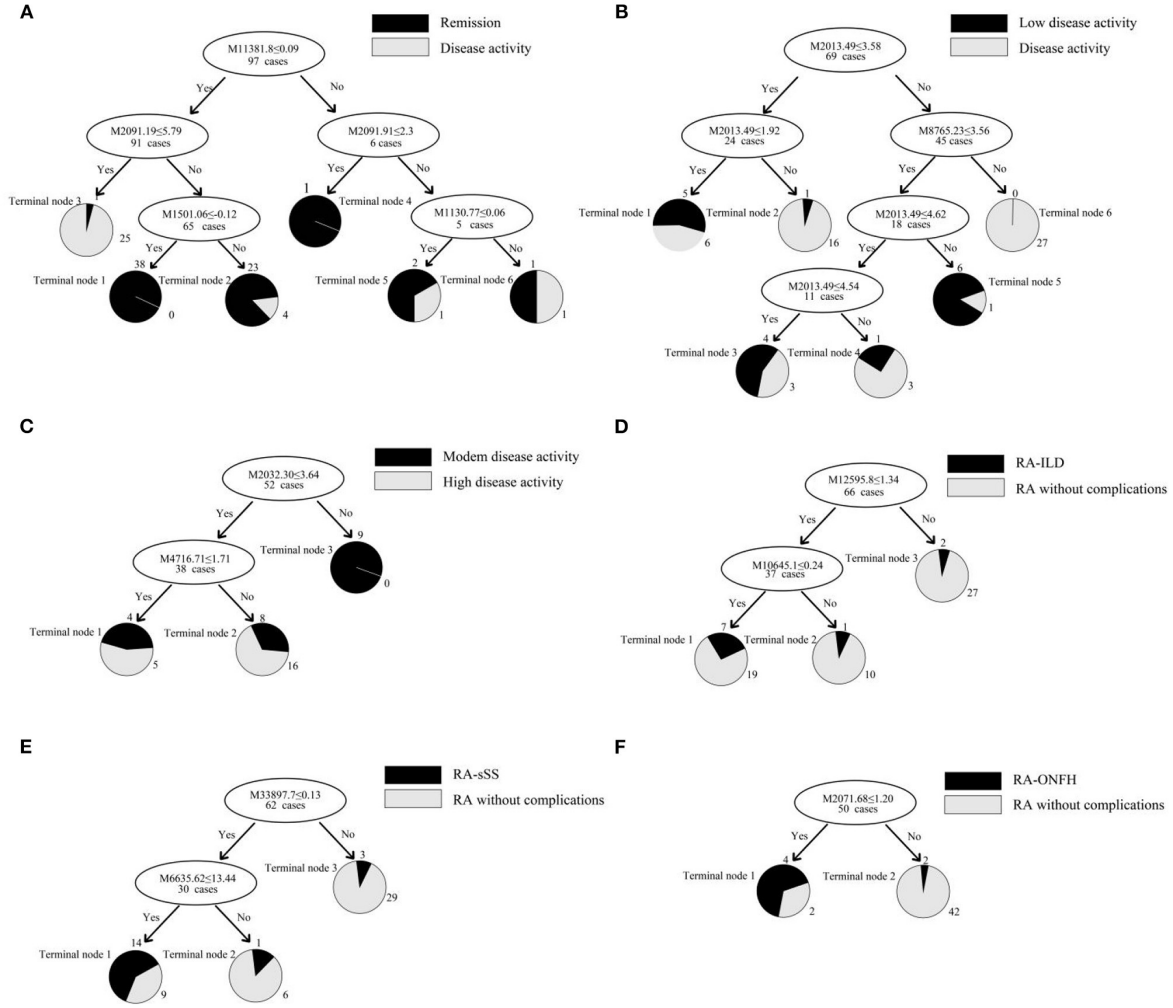
**(A–F)** Classification tree model for assessing disease activity in RA patients and distinguishing RA patients with complications. The mass-to-charge value in the nodes was followed by lower or equal to intensity value. If the answer to the question in a node of the tree was yes, it proceeded down to the left node, otherwise it proceeded down to the right node. When proceeding to the terminal nodes, the decision tree assigned to samples to different groups. **(A)**: patients with remission (terminal node 2, 4, and 5) and disease activity (terminal node 1, 3, and 6); **(B)**: RA patients with low disease activity (terminal node 1, 3, and 5) and other disease activity (terminal node 2, 4, and 6); **(C)**: RA patients with moderate disease activity (terminal node 2) and high disease activity (terminal node 1 and 3); **(D)**: RA-ILD (terminal node 1); **(E)**: RA-sSS (terminal node 1); **(F)**: RA-ONFH (terminal node 1).

**Figure 4:**
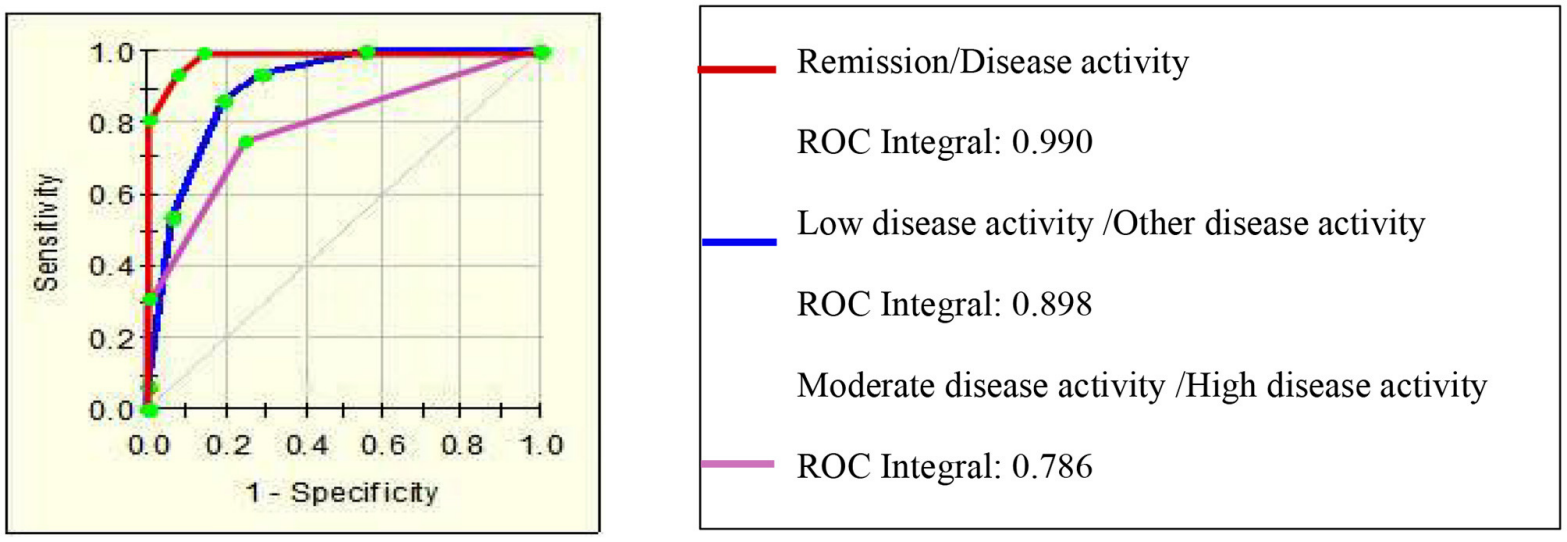
The ROC of the diagnostic models for assessing the activity of disease in RA patients.

This classification tree model was established from 28 RA patients with remission and 69 disease activity patients and had m/z 1,130.776, 1,501.065, 2,091.198, and 11,381.87 (Table 4) and six terminal nodes (Figure 3A). It had a sensitivity of 94.12% and a specificity of 93.33% (Table 5). The integral of ROC was 0.990 (Figure 4).

**Table 4:** Biomarker statistics for assessing the activity of disease in RA patients.

**M/Z**	**Remission (Mean ± SD)**	**Disease activity (Mean ± SD)**	***P***
1,130.78	1.01 ± 1.75	1.18 ± 1.54	0.013
1,501.70	1.56 ± 1.79	−0.19 ± 1.26	0.003
2,091.20	3.08 ± 1.34	4.52 ± 2.01	0.025
11,381.87	0.02 ± 0.16	0.16 ± 0.13	0.014
**M/Z**	**Low disease activity (Mean** **±** **SD)**	**Other disease activity (Mean** **±** **SD)**	***p***
2,013.49	3.71 ± 1.80	5.52 ± 2.59	0.019
8,765.23	4.25 ± 4.48	2.09 ± 1.91	0.026
**M/Z**	**Moderate disease activity (Mean** **±** **SD)**	**High disease activity (Mean** **±** **SD)**	***P***
2,032.31	1.57 ± 1.44	2.59 ± 1.95	0.047
8,214.28	2.65 ± 1.43	1.83 ± 1.35	0.042

**Table 5:** Establishment of the classification tree models for assessing the activity of disease in RA patients.

	**Classification tree model**	**Accuracy (%)**	**Sensitivity (%)**	**Specificity (%)**
Remission/ Disease activity(28/69)	M/Z1130.78, 1,501.07, 2,091.20, 11,381.87	92.78	94.12	93.33
Low disease activity/Other disease activity (17/52)	M2013.49, 8,765.23	82.61	86.67	80.65
Moderate disease activity/High disease activity (21/31)	M/Z2032.31, 4,716.71	75.00	74.19	76.19

#### RA Patients With Low Disease Activity and Other Disease Activity

This classification tree model was built from the serum samples of 17 RA patients with low disease activity and 52 patients with other disease activity and had m/z 2,032.31, 2,506.21, and 9,286.50 (Table 4) and five terminal nodes (Figure 3B). It had a sensitivity of 86.67% and a specificity of 80.65% (Table 5). The integral of ROC was 0.898 (Figure 4).

#### RA Patients With Moderate Disease Activity and High Disease Activity

This classification tree model was established to detect a distinction between RA patients with moderate disease activity and those with high disease activity and had m/z 2,032.31 and 4,716.71 (Table 4) and three terminal nodes (Figure 3C) It had a sensitivity of 74.19% and a specificity of 76.19% (Table 5). The integral of ROC was 0.786 (Figure 4).

### Discriminating m/z Peaks Between RA and RA With Complications (RA-ILD, RA-sSS, and RA-ONFH)

#### RA and RA-ILD

**Figure 5:**
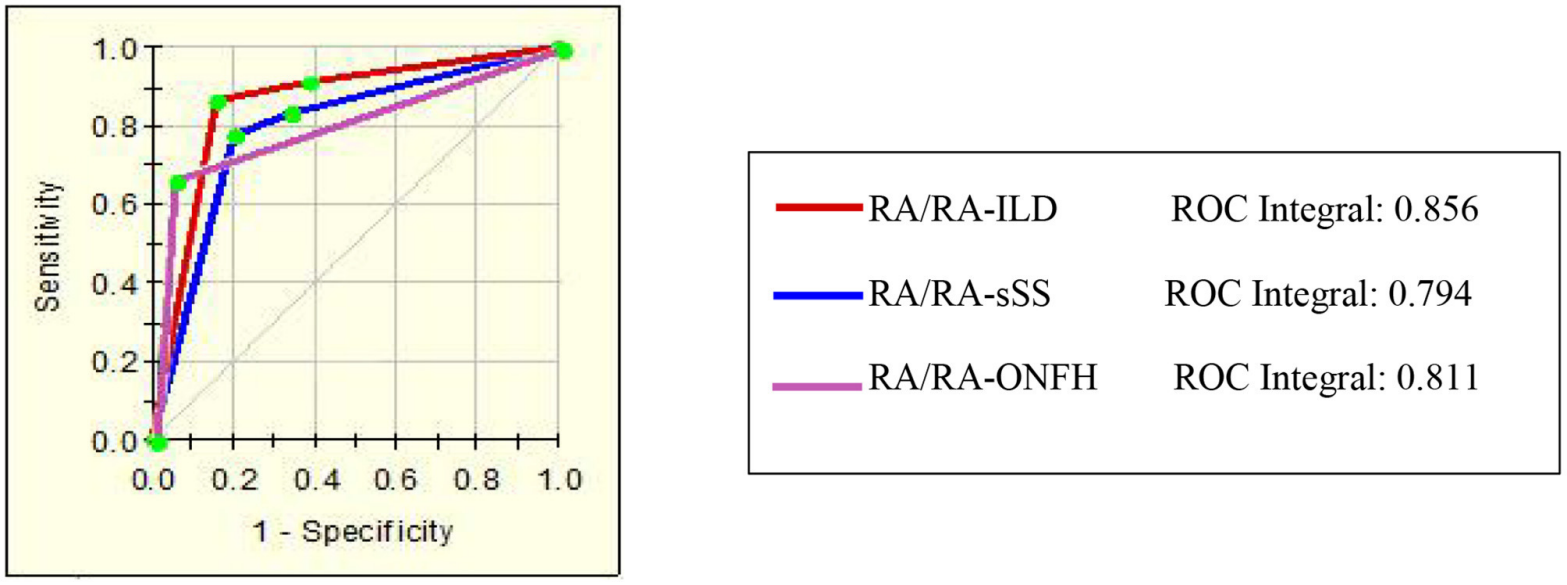
The ROC of the diagnostic models of RA with complications.

A total of 13 protein peaks from serum samples including 22 RA-ILD and 44 RA patients without complications were detected (*p* < 0.05) by MALDI-TOF-MS combined with WCX magnetic beads, which were down-regulated in patients with RA-ILD. We also used BPM5.0 to establish the most optimal tree model to distinguish between RA-ILD and RA without complications, which had m/z 10,645.10 and 12,595.86 (Table 6) and included three terminal nodes (Figure 3D). It has a sensitivity of 86.36% and a specificity of 84.09%, respectively (Table 7). The integral of ROC was 0.856 (Figure 5).

**Table 6:** Biomarker statistics for RA vs. RA with complications spectra in the decision tree classification.

**M/Z**	**RA + ILD (Mean ± SD)**	**RA (Mean ± SD)**	***P***
10,645.10	0.12 ± 0.36	0.47 ± 0.70	0.003
12,595.86	0.84 ± 0.44	1.55 ± 0.84	0
**M/Z**	**RA+sSS (Mean** **±** **SD)**	**RA (Mean** **±** **SD)**	***p***
6,635.62	11.38 ± 6.10	14.79 ± 5.89	0.045
33,897.72	0.11 ± 0.06	0.15 ± 0.05	0.037
**M/Z**	**RA+ONFH (Mean** **±** **SD)**	**RA (Mean** **±** **SD)**	***P***
2,071.69	1.73 ± 2.11	3.43 ± 1.63	0.025

**Table 7:** The classification tree models for RA and RA with complications.

	**Classification tree model**	**Accuracy**	**Sensitivity**	**Specificity**
RA/RA- ILD(44/22)	M/Z10645.10, 12,595.86	84.85%	86.36%	84.09%
RA/RA-sSS(44/18)	M/Z6635.62, 33,897.72	79.03%	77.78%	79.55%
RA/RA-ONFH(44/6)	M/Z2071.69	92.00%	66.67%	95.45%

#### RA and RA-sSS

Thirteen proteins from serum samples of 18 RA-sSS and 44 RA patients without complications were detected (*p* < 0.05). All were down-regulated in patients with RA-sSS. We also established the classification tree model by BPM5.0, which comprised m/z 6,635.62 and 33,897.72 (Table 6). The RA-sSS classification tree model included 3 terminal nodes (Figure 3E). After identification using the model, the patients were classified as having RA and RA-sSS with a specificity of 77.78% and a sensitivity of 79.55% (Table 7). The integral of ROC of this RA-sSS decision tree was 0.794 (Figure 5).

#### RA and RA-ONFH

There were two differential protein peaks/spot detected between 6 RA-ONFH and 44 RA patients without complications (*p* < 0.05). They were down-regulated in patients with RA-ONPH and were added into BPM5.0 to generate the classification tree model, which had m/z 2,071.69 (Table 6). The classification tree model included two terminal nodes (Figure 3F), which had a sensitivity of 66.67% and a specificity of 95.45% (Table 7). The integral of ROC was 0.811 (Figure 5).

### Developing Biological Markers for Etanercept Used in RA Patients

A total of four protein peaks were detected in RA patients between the responder (*n* = 7) and non-responder groups (*n* = 4) (*p* < 0.05), which included m/z 2,671.60, 5,801.84, 8,130.19, and 9,286.49. Three proteins at m/z 5,801.84, 8,130.19, and 9,286.49 were decreased in the responder group, whereas one at m/z 2,671.60 was overexpressed in the responder population.

## Discussion

We developed a classification tree model for early RA diagnosis by MALDI-TOF-MS with WCX magnetic beads, which included m/z 3,448.85, 4,716.71, 8,214.28, and 10,645.10. This diagnostic model was applied for RA and had a much higher specificity (91.566%) and sensitivity (92.857%). In addition, the classification tree model was tested among 14 RA patients and 20 HCs in blinded test set and had a sensitivity of 95.0% and a specificity of 100%. The four peaks might be critical peptides or proteins involved in the pathogenesis. Similar to the study by Zhang et al. (31), clinically useful biomarkers for RA were identified with this approach to discriminate patients with RA from HCs. Zhang et al. (32) reported that m/z 15,715.5, 7,771.4, 8,959.4, 8,469.8, and 8,710.8 in serum are of certain value for differential diagnosis of RA from osteoarthritis (OA) and HCs. Compared with these prior studies, we not only completed the building of the RA diagnostic model but also determined the origin and full identity of the biomarkers for RA diagnosis. We preliminarily retrieved components comprising these four protein peaks of diagnostic model from the ExPasy database, which was used to discriminate RA patients from the HCs. The four biomarkers may probably be CCL24, MT1DP, SLN, and CXCL-11, which were further identified and quantified through ELISA. Only the CCL24 level was detected to have a significant decrease in the serum of RA patients as compared with HCs (*p* < 0.05). No significant difference was found in others, but a decreasing trend consistent with the down-regulation of the four biomarkers detected by MALDI-TOF-MS was observed. The results agreed with previous reports. In Aloush et al. study, a significant decrease was observed in the level of CCR3 receptor in the serum of RA patients compared with HCs (33). CCL24/eotaxin-2 acts via highly specific activation of the CCR3 receptor. Another study observed that anti-eotaxin-2 antibody has a significant protective effect in adjuvant-induced arthritis in rats (34). Noticeably, a specific pattern was observed for mRNA expression in CCL24/eotaxin-2, which was higher among RA patients with lower X-ray scores than those with severe X-ray scores. The same results were also found in the comparison between the two groups with RA with low and medium disease activity and the subgroup with high activity (35). Combined with the above results, CCL24 may play a vital role in the pathogenesis of RA. The lower level of CCL24 in our result may be due to the reaction of eotaxin-2 to therapeutic manipulation in RA. However, in study by Uchida et al. (36), who detected discriminatory biomarkers to differentiate RA from OA using synovial fluid, they purified the protein based the detected specific peaks and the result revealed that m/z 10,850 was the clearest signal found specifically in RA and that it is myeloid-related protein 8, which might be related to the disease activity in RA. Despite the different results among research, these results highlighted that the CCL24, as a biomarker of this model, may be vital proteins or peptides that participate in the pathogenesis of RA and may be a promising potential therapeutic target for arthritis (37).

Although other biomarkers for the RA diagnostic model did not significantly differ in our result, the ELISA results, i.e., the four biomarkers had lower levels in RA patients than in HCs, were consistent with the MALDI-TOF-MS results. Thus far, MT1DP and SLN as the possible components, are not reported in studies about autoimmune disease. According to previous studies, the fourth protein, CXCL11, is believed to be the dominant CXCR3 agonist because it is more potent (38), and it has been believed to play a vital role in directing Th1 cells to sites of inflammation (39). Recent studies strongly concluded that the CXCR3 receptor is a potential therapeutic target for treating autoimmune diseases, such as RA and multiple sclerosis (40). Although we were not able to identify other biomarkers, possibly due to the relatively small sample of patients in this study, the possibility remains that this chemokine plays a vital role in RA.

We also aimed to construct a series of clinical models for an accurate assessment model of disease activity. Thus far, there are no studies about it, and we did not find relevant conclusions for the assessment model of RA in the present research. Consequently, we established the classification tree model for assessing the disease activity in RA patients. At first, we can use the classification model including m/z 1,130.78, 1,501.07, 2,091.20, and 11,381.87 to distinguish patients with disease activity from RA patients with remission. RA patients with low disease activity could be efficiently discriminated from other disease activity patients with the combined use of specific protein biomarkers (m/z 2,032.31, 2,506.21, and 9,286.50). Finally, m/z 2,032.31 and 4,716.71 were applied to build the classification model for RA patients with moderate disease activity and high disease activity. These models are possibly the most convenient and efficient clinical methods for RA patients.

After assessing and diagnosing RA patients using the established model, they should be comprehensively assessed to determine other complications, which can prompt early treatment. Therefore, we built the classification tree model for to accurately recognize RA-ILD, RA-sSS, and RA-ONFH from RA. The most optimal tree model to distinguish between RA-ILD and RA without complication had m/z 10,645.10, and 12,595.86. In the present research, the diagnostic model was applicable for RA-sSS and/or SS-ILD (41), but no biomarkers discriminating for RA-ILD were found. The RA-sSS model had m/z 6,635.62 and 33,897.72 and had a specificity of 77.78% and a sensitivity of 79.55%. In the study of Li et al. (42), m/z peaks at 8,133.85, 11,972.8, 2,220.81, and 4,837.66 were used to establish a diagnostic model for Primary Sjögren's syndrome from systemic lupus erythematosus, RA, and HCs. The m/z peaks of RA-sSS model were different among these studies. We believe that the difference might be due to the different characteristics of the subjects. Our study detected that m/z 2,071.68 has potential in discriminating between RA and RA-ONFH. A previous study (43) showed that seven proteins were found based on two-dimensional electrophoresis patterns from the sera of 10 patients with ONFH and 10 normal subjects. ELISA revealed that the levels of tissue plasminogen activator, plasminogen activator inhibitor 1, crosslaps, and anti-p53 antibody in patients with ONFH were always significantly different among patients with OA, RA, and fracture. In the diagnostic models of RA with complications, all had different protein peaks; thus, further research is needed to clarify its specific components.

The biological markers for etanercept used in RA patients were also generated in our study. A total of 4 protein peaks, including m/z 2,671.60, 5,801.84, 8,130.20, and 9,286.49, were detected in RA patients between the responder (*n* = 7) and non-responder groups (*n* = 4). The study of Trocme et al. (44) revealed factors predictive of infliximab therapeutic response. Five proteins at 3.86, 7.77, 7.97, 8.14, and 74.07 kDa were overexpressed in the non-responder group (*n* = 28), whereas one at 28 kDa was increased in the responder group (*n* = 32). Biomarker characterization showed that apolipoprotein A-1 was predictive of a good response to infliximab, whereas platelet factor four was associated with non-response, thereby providing some thoughts to the scientific research in our future study.

These data suggested that MALDI-TOF-MS combined with WCX magnetic beads could be helpful in clinical applications for efficient diagnosis and accurate assessment of disease activity, and it is useful for differentiating RA patients with complications, which plays an important role in providing early treatment to RA patients, thereby preventing worse symptoms in the future. Nevertheless, the identification of these biomarkers is essential in understanding the pathogenesis of RA, on which they may play a vital role. Therefore, our future study will complete the identification of other potential diagnostic biomarkers for RA, and their identification will further improve the usefulness of the model, shed further light on the pathogenesis of RA, and promote the study on the diagnostic biomarkers for RA.

In many health-care systems, the medical treatment of patients comprises three phases (clinical diagnosis, assessment, and treatment), to which the proposed models we established above are applied. Unlike findings about biomarkers discovered in previous studies, this study not only explored the diagnostic criteria established by proteomic technology to accurately recognize RA patients from HCs but also assessed the disease activity and developed classification tree models for comprehensive assessment of patient's condition and to determine whether they have other complications. Our study detected the potential biomarkers for etanercept used in RA patients, which can help solve future clinical problems. We further identified and quantified the biomarkers for RA and found that the CCL24 level was lower in the RA group than in HCs. In future study, we will complete the identification of other potential diagnostic biomarkers to explore their role in the pathogenesis and to detect the precise therapeutic target.

However, this study has some limitations. The tested sample size is small and should be increased. To identify the discovered protein peak, the sample size should be increased. Furthermore, no pathological controls with undifferentiated arthritis, OA, or inflammatory joint diseases were assayed in this study. Thus, these should be considered in future research. More effort in future studies should be done, including increasing the sample size and completing other molecular identification of the potential specific biomarkers reported in this study for a better understanding of RA.

## Conclusion

Our study successfully established a series of specific proteomic diagnostic models for RA, RA-ILD, RA-sSS, and RA-ONFH and detected the potential biomarkers to distinguish RA disease activity and to identify etanercept's clinical effect using proteomic fingerprint technology (MALDI-TOF-MS) combined with WCX magnetic beads. Furthermore, we have also demonstrated the differential expression of CCL24 in RA serum and HCs, which appears to have a pathogenetic role in RA and may serve as therapeutic targets in the future. Further exploration of these findings requires a larger sample size.

## Data Availability Statement

The raw data supporting the conclusions of this article will be made available by the authors, without undue reservation.

## Ethics Statement

The studies involving human participants were reviewed and approved by Ethics Committee of the Shanxi Bethune Hospital. The patients/participants provided their written informed consent to participate in this study.

## Author Contributions

LZ: study concept and design. DM, NL, and LZ: acquisition, analysis and interpretation of data, statistical analysis, drafting of the manuscript, and critical revision of the manuscript. All authors read and approved the final manuscript.

## Conflict of Interest

The authors declare that the research was conducted in the absence of any commercial or financial relationships that could be construed as a potential conflict of interest.

## References

[1] McInnes I Schett G. The pathogenesis of rheumatoid arthritis. N Engl J Med. (2011) 365:2205–19. 10.1056/NEJMra1004965 22150039

[2] Turesson C O'Fallon W Crowson C Gabriel S Matteson E. Extra-articular disease manifestations in rheumatoid arthritis: incidence trends and risk factors over 46 years. Ann Rheum Dis. (2003) 62:722–7. 10.1136/ard.62.8.722 12860726 PMC1754626

[3] Spagnolo P Lee J Sverzellati N Rossi G Cottin V. The Lung in rheumatoid arthritis: focus on interstitial lung disease. Arthritis Rheumatol. (2018) 70:1544–54. 10.1002/art.40574 29806092

[4] Gochuico B Avila N Chow C Novero L Wu H Ren P. Progressive preclinical interstitial lung disease in rheumatoid arthritis. Arch Intern Med. (2008) 168:159–66. 10.1001/archinternmed.2007.59 18227362

[5] Hajiabbasi A Shenavar Masooleh I Alizadeh Y Banikarimi A Ghavidel Parsa P. Secondary Sjogren's syndrome in 83 patients with rheumatoid arthritis. Acta Med Iran. (2016) 54:448–53. 27424016

[6] Gilboe I Kvien T Uhlig T Husby G. Sicca symptoms and secondary Sjögren's syndrome in systemic lupus erythematosus: comparison with rheumatoid arthritis and correlation with disease variables. Ann Rheum Dis. (2001) 60:1103–9. 10.1136/ard.60.12.1103 11709451 PMC1753445

[7] Weinstein R. Clinical practice. Glucocorticoid-induced bone disease. N Engl J Med. (2011) 365:62–70. 10.1056/NEJMcp1012926 21732837

[8] Watanabe Y Kawai K Hirohata K. Histopathology of femoral head osteonecrosis in rheumatoid arthritis: the relationship between steroid therapy and lipid degeneration in the osteocyte. Rheumatol Int. (1989) 9:25–31. 10.1007/bf00270286 2772484

[9] Mun S Lee J Park A Kim H Lee Y Son H. Proteomics approach for the discovery of rheumatoid arthritis biomarkers using mass spectrometry. Int J Mol Sci. (2019) 20:4368. 10.3390/ijms20184368 31491989 PMC6769564

[10] Myasoedova E Davis J Matteson E Crowson C. Is the epidemiology of rheumatoid arthritis changing? Results from a population-based incidence study, 1985-2014. Ann Rheum Dis. (2020) 79:440–4. 10.1136/annrheumdis-2019-216694 32066556 PMC7085464

[11] Travis W Costabel U Hansell D King T Lynch D Nicholson A. An official American Thoracic Society/European Respiratory Society statement: update of the international multidisciplinary classification of the idiopathic interstitial pneumonias. Am J Respir Crit Care Med. (2013) 188:733–48. 10.1164/rccm.201308-1483ST 24032382 PMC5803655

[12] Assayag D Elicker B Urbania T Colby T Kang B Ryu J. Rheumatoid arthritis-associated interstitial lung disease: radiologic identification of usual interstitial pneumonia pattern. Radiology. (2014) 270:583–8. 10.1148/radiol.13130187 24126367 PMC4228744

[13] Saini A Saifuddin A. MRI of osteonecrosis. Clin Radiol. (2004) 59:1079–93. 10.1016/j.crad.2004.04.014 15556590

[14] Jones L Hungerford D. Osteonecrosis: etiology, diagnosis, and treatment. Curr Opin Rheumatol. (2004) 16:443–9. 10.1097/01.moo.0000127829.34643.fd 15201609

[15] Baldini C Talarico R Tzioufas A Bombardieri S. Classification criteria for Sjogren's syndrome: a critical review. J Autoimmun. (2012) 39:9–14. 10.1016/j.jaut.2011.12.006 22209352

[16] Kyriakidis N Kapsogeorgou E Tzioufas A. A comprehensive review of autoantibodies in primary Sjögren's syndrome: clinical phenotypes and regulatory mechanisms. J Autoimmun. (2014) 51:67–74. 10.1016/j.jaut.2013.11.001 24333103

[17] Gremese E Salaffi F Bosello S Ciapetti A Bobbio-Pallavicini F Caporali R. Very early rheumatoid arthritis as a predictor of remission: a multicentre real life prospective study. Ann Rheum Dis. (2013) 72:858–62. 10.1136/annrheumdis-2012-201456 22798566 PMC3664395

[18] Tsuchida S Umemura H Nakayama T. Current status of Matrix-Assisted Laser Desorption/Ionization-Time-of-Flight Mass Spectrometry (MALDI-TOF MS) in clinical diagnostic microbiology. Molecules. (2020) 25:4775. 10.3390/molecules25204775 33080897 PMC7587594

[19] Angeletti S Ciccozzi M. Matrix-assisted laser desorption ionization time-of-flight mass spectrometry in clinical microbiology: an updating review. Infect Genet Evol. (2019) 76:104063. 10.1016/j.meegid.2019.104063 31618693

[20] Baumann S Ceglarek U Fiedler G Lembcke J Leichtle A Thiery J. Standardized approach to proteome profiling of human serum based on magnetic bead separation and matrix-assisted laser desorption/ionization time-of-flight mass spectrometry. Clin Chem. (2005) 51:973–80. 10.1373/clinchem.2004.047308 15845803

[21] Whiteaker J Zhao L Zhang H Feng L Piening B Anderson L. Antibody-based enrichment of peptides on magnetic beads for mass-spectrometry-based quantification of serum biomarkers. Anal Biochem. (2007) 362:44–54. 10.1016/j.ab.2006.12.023 17241609 PMC1852426

[22] Peter J Otto A. Magnetic particles as powerful purification tool for high sensitive mass spectrometric screening procedures. Proteomics. (2010) 10:628–33. 10.1002/pmic.200900535 20099258

[23] Li Y Hu C Leng X Zhao G Li N Xu Y. Promising diagnostic biomarkers for primary biliary cirrhosis identified with magnetic beads and MALDI-TOF-MS. Anat Rec. (2009) 292:455–60. 10.1002/ar.20870 19248174

[24] Goëb V Thomas-L'Otellier M Daveau R Charlionet R Fardellone P Le Loët X. Candidate autoantigens identified by mass spectrometry in early rheumatoid arthritis are chaperones and citrullinated glycolytic enzymes. Arthritis Res Ther. (2009) 11:R38. 10.1186/ar2644 19284558 PMC2688184

[25] de Seny D Fillet M Meuwis M Geurts P Lutteri L Ribbens C. Discovery of new rheumatoid arthritis biomarkers using the surface-enhanced laser desorption/ionization time-of-flight mass spectrometry ProteinChip approach. Arthritis Rheum. (2005) 52:3801–12. 10.1002/art.21607 16320331

[26] Huang Z Shi Y Cai B Wang L Wu Y Ying B. MALDI-TOF MS combined with magnetic beads for detecting serum protein biomarkers and establishment of boosting decision tree model for diagnosis of systemic lupus erythematosus. Rheumatology (Oxford). (2009) 48:626–31. 10.1093/rheumatology/kep058 19389822

[27] Giusti L Baldini C Bazzichi L Bombardieri S Lucacchini A. Proteomic diagnosis of Sjögren's syndrome. Expert Rev Proteomics. (2007) 4:757–67. 10.1586/14789450.4.6.757 18067414

[28] Lin X Yang S Du J Tian Y Bu L Huo S. Detection of lung adenocarcinoma using magnetic beads based matrix-assisted laser desorption/ionization time-of-flight mass spectrometry serum protein profiling. Chin Med J. (2010) 123:34–9. 10.3760/cma.j.issn.0366-6999.2010.01.006 20137572

[29] Li J Zhang Z Rosenzweig J Wang Y Chan D. Proteomics and bioinformatics approaches for identification of serum biomarkers to detect breast cancer. Clin Chem. (2002) 48:1296–304. 10.1016/S0009-8981(02)00170-5 12142387

[30] Cheng A Chen L Chien K Chen Y Chang J Wang H. Oral cancer plasma tumor marker identified with bead-based affinity-fractionated proteomic technology. Clin Chem. (2005) 51:2236–44. 10.1373/clinchem.2005.052324 16239339

[31] Yan Z Chaojun H Chuiwen D Xiaomei L Xin Z Yongzhe L. Establishing serological classification tree model in rheumatoid arthritis using combination of MALDI-TOF-MS and magnetic beads. Clin Exp Med. (2015) 15:19–23. 10.1007/s10238-013-0265-2 24292670

[32] Zhang X Yuan Z Shen B Zhu M Liu C Xu W. Discovery of serum protein biomarkers in rheumatoid arthritis using MALDI-TOF-MS combined with magnetic beads. Clin Exp Med. (2012) 12:145–51. 10.1007/s10238-011-0154-5 21922190

[33] Aloush V George J Elkayam O Wigler I Oren S Entin-Meer M. Decreased levels of CCR3 in CD4+ lymphocytes of rheumatoid arthritis patients. Clin Exp Rheumatol. (2010) 28:462–7. 10.1186/1471-2474-11-143 20659406

[34] Ablin J Entin-Meer M Aloush V Oren S Elkayam O George J. Protective effect of eotaxin-2inhibition in adjuvant-induced arthritis. Clin Exp Immunol. (2010) 161:276–83. 10.1111/j.1365-2249.2010.04172.x 20456418 PMC2909409

[35] Zhebrun D Totolyan A Maslyanskii A Titov A Patrukhin A Kostareva A. Synthesis of some CC chemokines and their receptors in the synovium in rheumatoid arthritis. Bull Exp Biol Med. (2014) 158:192–6. 10.1007/s10517-014-2720-9 25430645

[36] Uchida T Fukawa A Uchida M Fujita K Saito K. Application of a novel protein biochip technology for detection and identification of rheumatoid arthritis biomarkers in synovial fluid. J Proteome Res. (2002) 1:495–9. 10.1021/pr025531w 12645617

[37] Cole K Strick C Paradis T Ogborne K Loetscher M Gladue R. Interferon-inducible T cell alpha chemoattractant (I-TAC): a novel non-ELR CXC chemokine with potent activity on activated T cells through selective high affinity binding to CXCR3. J Exp Med. (1998) 187:2009–21. 10.1084/jem.187.12.2009 9625760 PMC2212354

[38] Karin N Razon H. Chemokines beyond chemo-attraction: CXCL10 and its significant role in cancer and autoimmunity. Cytokine. (2018) 109: 24–8. 10.1016/j.cyto.2018.02.012 29449068

[39] C. Jenh, M. Cox, L. Cui, E. Reich, L. Sullivan, S. Chen, et al. A selective and potent CXCR3 antagonist SCH 546738 attenuates the development of autoimmune diseases and delays graft rejection. BMC immunology. (2012) 13:2. 10.1186/1471-2172-13-2 22233170 PMC3298469

[40] Korniejewska A McKnight A Johnson Z Watson M Ward S. Expression and agonist responsiveness of CXCR3 variants in human T lymphocytes. Immunology. (2011) 132:503–15. 10.1111/j.1365-2567.2010.03384.x 21255008 PMC3075504

[41] Li Y Sun X He J Jia R Yang D Zhang X. Screening for serum specific biomarkers in patients with primary Sjögren's syndrome and interstitial lung disease using proteomic fingerprint techniques. Beijing Da Xue Xue Bao. (2012) 44:240–3. 10.3969/j.issn.1671-167X.2012.02.017 22516996

[42] Li Y Sun X Zhang X Yang Y Jia R Liu X. Establishment of a novel diagnostic model for Sjögren's syndrome by proteomic fingerprinting. Clin Rheumatol. (2014) 33:1745–50. 10.1007/s10067-014-2762-4 25178777

[43] Tan X Cai D Wu Y Liu B Rong L Chen Z. Comparative analysis of serum proteomes: discovery of proteins associated with osteonecrosis of the femoral head. Transl Res. (2006) 148:114–9. 10.1016/j.trsl.2006.05.001 16938648

[44] Trocmé C Marotte H Baillet A Pallot-Prades B Garin J Grange L. Apolipoprotein A-I and platelet factor 4 are biomarkers for infliximab response in rheumatoid arthritis. Ann Rheum Dis. (2009) 68:1328–33. 10.1136/ard.2008.093153 18664547 PMC2921545

